# Extended Reality-Based Proof-of-Concept for Clinical Assessment Balance and Postural Disorders for Personalized Innovative Protocol

**DOI:** 10.3390/bioengineering12080850

**Published:** 2025-08-07

**Authors:** Fabiano Bini, Michela Franzò, Alessia Finti, Francesca Tiberi, Veronica Maria Teresa Grillo, Edoardo Covelli, Maurizio Barbara, Franco Marinozzi

**Affiliations:** 1 Department of Mechanical and Aerospace Engineering, Sapienza University of Rome, 00184 Rome, Italy; alessia.finti@uniroma1.it (A.F.); franco.marinozzi@uniroma1.it (F.M.); 2 Department of Psychology, Sapienza University of Rome, 00185 Rome, Italy; michela.franzo@uniroma1.it; 3 Department of NESMOS, Otolaryngology Clinic, Faculty of Medicine and Psychology, Sapienza University of Rome, 00189 Rome, Italyedoardo.covelli@uniroma1.it (E.C.); maurizio.barbara@uniroma1.it (M.B.)

**Keywords:** extended reality, balance assessment, balance disorders, vestibular pathology, rehabilitation

## Abstract

Background: Clinical assessment of balance and postural disorders is usually carried out through several common practices including tests such as the Subjective Visual Vertical (SVV) and Limit of Stability (LOS). Nowadays, several cutting-edge technologies have been proposed as supporting tools for stability evaluation. Extended Reality (XR) emerges as a powerful instrument. This proof-of-concept study aims to assess the feasibility and potential clinical utility of a novel MR-based framework integrating HoloLens 2, Wii Balance Board, and Azure Kinect for multimodal balance assessment. An innovative test is also introduced, the Innovative Dynamic Balance Assessment (IDBA), alongside an MR version of the SVV test and the evaluation of their performance in a cohort of healthy individuals. Results: All participants reported SVV deviations within the clinically accepted ±2° range. The IDBA results revealed consistent sway and angular profiles across participants, with statistically significant differences in posture control between opposing target directions. System outputs were consistent, with integrated parameters offering a comprehensive representation of postural strategies. Conclusions: The MR-based framework successfully delivers integrated, multimodal measurements of postural control in healthy individuals. These findings support its potential use in future clinical applications for balance disorder assessment and personalized rehabilitation.

## 1. Introduction

Maintaining balance is essential for the habitual activities performed by a human being. The alteration of the ability to maintain this state can occur following illness or physiological aging. Balance is also an essential skill for sports and exercise, and it requires accurate assessment in various fields like physiotherapy, sports science, geriatrics, and neurology [1]. Postural control is defined as the act of maintaining, achieving, or restoring a state of balance during any posture or activity [2].

The alteration in balance occurs when the vertical projection of the center of mass (CoM) moves beyond the limits of the base of support (BoS). This misalignment is encouraged daily by the exposure of the individual to external forces, leading to the need to continuously restore and maintain the condition of balance [3]. In particular, the degeneration of balance conditions in the elderly is one of the main symptoms related to aging. Aging and age-related disorders like strokes, traumatic brain injury (TBI), and Parkinson’s disease are closely linked to a variety of physiological changes that affect the motor, sensory, and cognitive systems [4].

The assessment of balance can be important for various patients, including those with neurological deficits, orthopedic deficits, and vestibular disorders. However, as highlighted by [4], the risk associated with balance problems, such as falling and reduced mobility, can be mitigated by early detection and the prompt execution of effective interventions.

According to [5], clinical balance assessment can be divided into three main approaches. The first approach is the functional assessment, which consists of verifying the existence of a balance disorder leading to the risk of falling. Secondly, the systems assessment focuses on researching the specific causes and solutions for balance disorders. Furthermore, it aims at identifying the subcomponents affecting the control of balance, such as biomechanics, motor coordination, and sensory organization. Finally, various types of quantitative posturography can be used to assess both causes and disease.

The evaluation of balance skills lacks a standardized approach universally recognized, resulting in a wide range of practices. Some conventional assessment tests used to evaluate balance skills are the Berg Balance Scale, Tinetti Balance Test, Romberg Test, and two slightly newer approaches, which are the Balance Evaluation Systems Test (BESTest) and the Limits of Stability (LOS) test [1]. The review conducted by Hanim revealed that conventional techniques are affected by limitations in capturing subtle aspects of balance control, and they require manual observation or specialist training to administer. The LOS is a test to explore the mechanical stability limits in erect stance [6]. Different software programs have been proposed to perform the LOS test, and some studies [7,8,9] have used them to evaluate the validity and reliability of the test. A simple and reliable test for assessing vestibular disorders is the ‘bucket test’; thus, the Subjective Visual Vertical (SVV) test [10] is a clinical examination that traditionally uses a bucket to assess an individual’s subjective perception of vertical orientation.

Sensor-based assessments [11] can potentially offer higher precision and a richer data profile, even if they are more complex and expensive. Therefore, complementing traditional clinical tests with technological tools like wearable sensors, force platforms, or motion capture systems may improve the accuracy and richness of balance evaluations.

Over the last 15 years, wearable devices have been used largely for the assessment of balance in patients affected by neurological disorders, providing valuable data compared with standard laboratory instrumentation [12]. Indeed, great experience in the use of wireless sensors for balance evaluation has been achieved in the laboratory setting, but much still needs to be done for the technological migration to the unsupervised domestic environment. The review conducted by [13] about the validity and reliability of dynamic and functional balance tests in people in the age range of 19–54 revealed that the Nintendo Wii Balance Board, Clever Balance Board, and Posturomed devices showed excellent reliability for assessing dynamic postural balance. Motion-capture systems are also conventionally used in balance assessment laboratories, though this field lacks low-cost and easy-to-use methods for clinical settings. However, in different fields, the accuracy and reliability of Azure Kinect for markerless tracking of body movements during these tasks were successfully validated against a marker-based motion capture system [14].

Among the useful technology for balance and training, Extended Reality (XR) emerges as a powerful instrument. Several studies [15] aim at validating the integration of Head-Mounted Display (HMD) with traditional methods to analyze the vestibular function through precise diagnostic values.

XR covers different technologies such as Augmented Reality (AR), Virtual Reality (VR), and Mixed Reality (MR). AR allows for a view of the real world—physical world—with an overlay of digital elements. VR is a fully immersive digital environment that isolates the user from the surroundings. MR still allows for a view of the physical world but with an overlay of digital elements where physical and digital elements can interact.

The study conducted by [16] revealed that VR with HMD influences head-level sway velocity, which correlates with increased visual disturbance, suggesting its potential as a low-risk standalone posturography tool. Moreover, VR and AR turned out to be useful for balance training in older adults [17,18] since the technologies appeared to stimulate increased motivation, a prerequisite for adherence to training. Finally, MR interventions turned out to have a positive effect on physical functions in the elderly [19] as rehabilitative [20,21,22] or assistive technology [23,24].

A previous integration of AR in clinical application was already presented in [25] for Dynamic Gait Index (DGI). DGI is a clinical measure commonly used to assess the ability to adapt gait to the complex walking tasks commonly encountered in daily life. During the test, eight elements are analyzed: walking on a flat surface, changing speed, turning the head horizontally and vertically, walking and turning 180 degrees, stepping over and around obstacles, and climbing and descending stairs. In the healthcare institutions where this test is conducted, optoelectronic and sensor-based acquisition systems are not currently implemented; therefore, the evaluation is performed exclusively qualitatively, based on the observation of the patient during the different phases of the test. When the exercise is performed wearing HMD, the obstacles are holographic objects and the headset allows the recording of time of execution, 3D coordinates, and 3D angles of the head and eyes.

Despite the variety of tools available, clinical balance assessment often lacks integration and ecological validity, especially in real-time, immersive environments. Recent studies suggest that sensor-based technologies and virtual environments may improve diagnostic accuracy, but they often rely on expensive setups or systems with limited patient interaction.

To address this gap, we present a novel proof-of-concept platform that integrates Microsoft HoloLens 2, Azure Kinect, and the Nintendo Wii Balance Board into a unified Mixed Reality (MR) environment for real-time, multimodal balance assessment. This study introduces and evaluates two immersive exercises: a reconfigured Subjective Visual Vertical (SVV) test and a new Innovative Dynamic Balance Assessment (IDBA), both designed to capture head position, CoP, and full-body kinematics simultaneously. The objective of this work is to assess the feasibility of this system in a healthy population, laying the groundwork for future clinical applications in rehabilitation and diagnosis.

Since XR has the potential to help users perform physical exercises that could improve their health conditions, the present study proposes a unique and integrated XR framework for balance disorder assessment that consists of a combination of the MR-HMD HoloLens 2 (HL2) (Microsoft), the Nintendo Wii Board for Center of Pressure (CoP) evaluation, and the Azure Kinect Technology for body joints tracking. Compared with existing methods, the system allows for detailed measurement not only of traditional balance parameters, such as the sway area of the CoP, but also of new integrative variables. In fact, the XR environment has been used to develop a new concept of the exercises SVV and LOS. The presented embedded system aims to propose a new protocol for the valuation of the global vision of the posture of the subject while reaching targets with their CoP. The exercise proposed is designed to be personalized after the traditional LOS and to be used for rehabilitative purposes or for assessing postural stability. Finally, the prototypes have been tested on a group of healthy volunteers.

This study aims to assess the feasibility and performance of multimodal balance assessment through MR. It fills a gap in the current literature by introducing and testing a novel MR platform that synchronously acquires head, joint, and CoP data using accessible technologies, aiming to inform future personalized rehabilitation protocols.

## 2. Materials and Methods

### 2.1. Hardware and Software

The HMD device used for the execution of the XR exercises is Microsoft HL2, and it is connected to a central computer with 32 GB RAM, Intel (Intel Corporation, Santa Clara, CA, USA) i7-1050H CPU 2.60 GHz, and an Internal Intel Video BIOS UHD graphic. Unity engine (2020.3.30f1) together with the MRTK 2.7 Microsoft Mixed Reality toolkit package allows the development and management of the MR scenarios through C# scripts. The exercises are performed using Holographic Remoting function, which allows holographic content to be streamed from a PC to Microsoft HoloLens in real time using a USB cable. Since HL2 can record just the head, hand, and eye tracking data, the Azure Kinect V3 tool (Microsoft, Albuquerque, NM, USA) is used to monitor the position of body joints during exercises, thereby ensuring a comprehensive view for conducting more in-depth research. The device needs no calibration process and can capture the movement following the Time-of-Flight distance principle between subject and device since it integrates an IR camera, a RGB camera, and an IMU (Inertial Measurement Unit) sensor. Information about the CoP is acquired through the Nintendo Wii Balance Board (WBB) device, which can measure vertical forces at the four corner edges of its surface owing to four uniaxial force transducers located at the corners. The software ‘Wii Balance Walker’ (Nintendo, Kyoto, Japan) and ‘BrainBLoX’ (Neuromechanics Laboratory—Department of Integrative Physiology—University of Colorado) are used to connect the WBB to the computer, record CoP data, and use it as a joystick, giving the patient the ability to control a fictional character on the MR scene. These software programs also include built-in calibration processes that are automatically executed before their use. Eventually, MATLAB (MathWorks, Natick, MA, USA) environment is used for data processing.

All devices are connected to a central PC where Unity Engine manages real-time communication between the HL2 (for head and gaze tracking), Azure Kinect (for skeletal joint detection), and the WBB (for CoP data). Synchronization across devices is achieved using Unity’s internal clock and event-driven acquisition scripts, ensuring alignment between holographic events and sensor recordings. Given the feasibility nature of the study, the evaluation of system latencies or desynchronization has not been evaluated yet.

### 2.2. Description of Balance Assessment Exercises in MR

#### 2.2.1. Innovative Dynamic Balance Assessment

The Innovative Dynamic Balance Assessment (IDBA) is an AR exercise designed with an intuitive and engaging nature with a gaming component. The exercise involves the WBB to control a character through CoP movements and the Azure Kinect device for the detection of body joints. HL2 allows the user to see the holographic game and records head tracking data (three-coordinates positions and three-axis angles). While traditional parameters such as sway area, CoP velocity, and trunk oscillations are well-established measures of postural control, the innovation of the IDBA lies in its simultaneous integration of multiple sensor modalities. Unlike previous approaches relying solely on CoP data, the IDBA combines WBB-based CoP measures with inertial sensor data recording trunk and hip angular oscillations. The multimodal integration permits more comprehensive balance dynamics estimation since it also tracks segmental motor control strategies not attainable with CoP analysis.

During the test, the patient would be standing on the WBB and asked to move their body weight in varied directions within their balance limits. The patient should move the CoP as a joystick to move a character on the screen toward the targets, which appear one at a time. The character moves in the platform according to the translation of the CoP in the horizontal and vertical axis recorded by the WBB in each instant. Knowing that the frequency of the Unity elaboration (around 30 Hz) is lower than the frequency of acquisition of the WBB (Nintendo, Kyoto, Japan) software (around 80 Hz), a velocity of translation is provided to the character to move from one position to another to simulate a more realistic movement of the character. So, the character moves according to Equation (1):

(1) $$\vec{S}=\hat{n}\times \left(v\times \Delta t\right)$$

where v is the velocity (set constant), ∆t is the time passed from the last frame acquired, n is the normal vector of the direction of translation of the CoP in the plane, and S is the translation of the character in the plane. Positions of the targets are set on the extreme border of the BoS to make the users reach their limit in maintaining balance.

**Figure 1:**
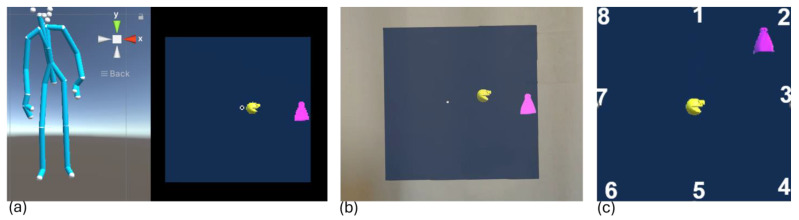
(**a**) Screenshot of the Unity platform during IDBA where on the left the skeleton of the use reconstructed by the Kinect in real-time is shown while on the right the visual of the participant from the headset is reported; (**b**) screenshot of the HL2 point of view; (**c**) eight positions where the targets appear and the initial position of Pac-Man. In all three images Pac-man is the yellow sphere and represent the position of the CoP of the participant, while the target to be reached is the violet ghost.

Through the HL2, the patient sees a platform of a game fixed in front of them and a yellow Pac-Man at the center. Pac-Man is the character that follows the movements of the patient’s CoP. As soon as the exercise begins, a row of pink ghosts appears in a radial pattern, indicating the targets the patient needs to shoot using Pac-Man (Figure 1). The exercise is designed after the ‘Vestlab test’ to be performed after the traditional LOS test. All targets appear in sequence, and each has a limited time of 10 s within which it must be reached. At the end of this time range, the target disappears, and the next one appears. Between the two targets, a ghost appears at the center of the platform with a limited time of 8 s. The aim of this section of the game is to make the patient assume the upright position again before chasing the next target.

**Figure 2:**
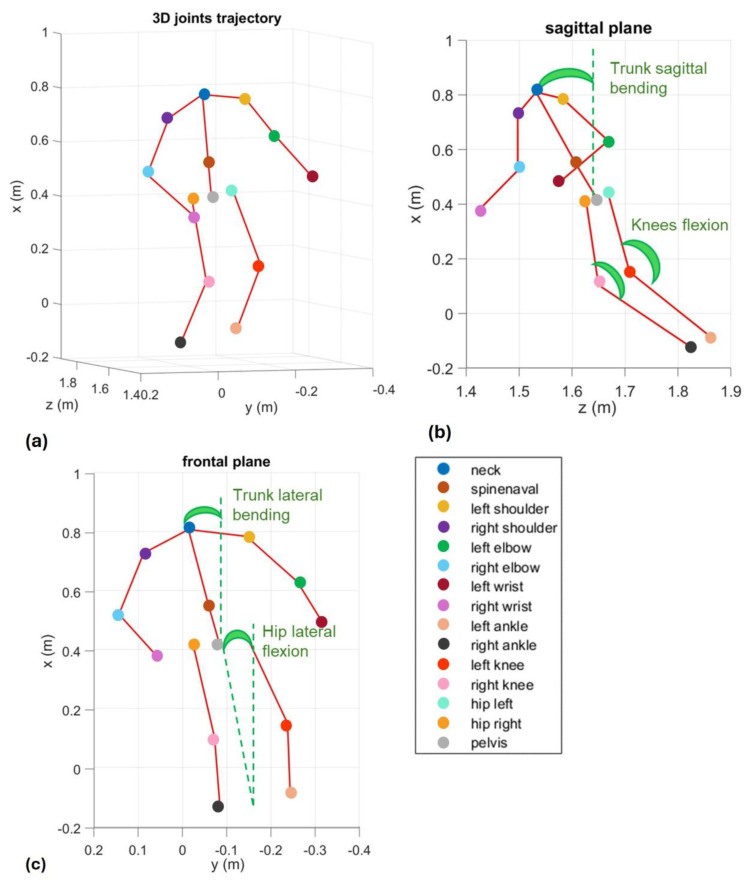
Representation of the angles calculated with the joints of the skeleton acquired through Azure Kinect Device in a single frame of time: (**a**) representation in the 3D space; (**b**) projection on the sagittal plane; (**c**) projection on the frontal plane. Kinect operates optimally within ~1.5 m range, and the 1 m reference in the figure relates to body segmentation scaling.

The subject is free to move the body to make the CoP reach the target. The purpose of this exercise is to acquire the posture that participants intuitively assume when performing the exercise and evaluate how they balance their bodies in the same situation. The posture of the subject is assessed through the estimation of specific angles starting from the joint’s positions. The angles suggested by the clinicians for a global posture analysis are knee flexions, trunk flexion on frontal and sagittal planes, and hip flexion on the frontal plane (Figure 2).

A video of the experience recorded from the HL2 point of view is presented in Video S1. Furthermore, a video recorded from the Unity engine platform Scene panel is presented in Video S2. The data collected by the prototype allows calculation of all the LOS parameters, and, through the introduction of HL2 and Azure Kinect, new parameters related to head positions and global posture. The parameters calculated for this study are reported in Table 1.

#### 2.2.2. Subjective Visual Vertical

**Figure 3:**
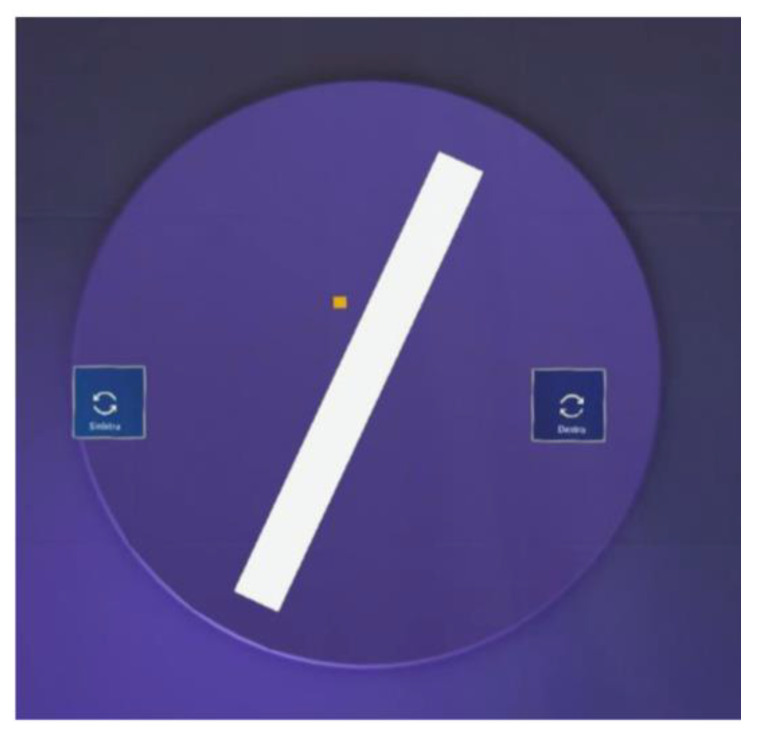
Screenshots of the HL2 point of view during SVV. The white bar is in the center of the participant’s view with a violet background to limit the vision, while two holographic buttons allow turning the white bar to the right and to the left. The yellow cube indicates the direction of the participant’s gaze.

The MR Subjective Visual Vertical (SVV) exercise requires only the use of the HL2, through which the user will see the holograms shown in Figure 3 and interact with them. A blue semitransparent cylinder is shown to limit the patient’s range of vision, and a white bar is displayed at the center. Initially, the white bar rotates automatically at a fixed velocity, and after some seconds, it stops at a certain inclination. The patient should reposition the white bar vertically using either voice commands or the two side buttons indicating rotation to the right or left. The bar can rotate 1 degree at a time.

As soon as the patient believes that the bar is positioned vertically, the exercise is stopped, and the data is saved. The angular inclinations of the position are measured in degrees and defined as positive deviations clockwise and negative counterclockwise. In the scientific literature, any deviation greater than ±2 degrees is considered a sign of pathology [10]. A video of the experience recorded from the HL2 point of view is presented in Video S3.

### 2.3. Data Analysis on a Control Group of Healthy Participants

#### 2.3.1. Experimental Setup and Protocol

This study was designed as an initial feasibility assessment in a healthy control population to determine system reliability and establish normative baseline metrics prior to extending to clinical populations with balance impairments. A group of 15 healthy participants (average age 27.11 ± 3.03 years old) volunteered to perform both SVV and LOS tests. The participants declared not reporting a history of motor impairments or balance disorders, and participants also provided informed consent before the experiment. Participants were informed of the tasks and allowed one trial for familiarization. Each participant performed the LOS and the SVV tests. Tests were administered in a random order to avoid sequence effects. Experiments were conducted in the laboratory of Industrial Bioengineering at Sapienza University of Rome. The experimental setup included the HL2 for head tracking, the WBB for CoP measurements, and the Azure Kinect device to record joint angles. The devices were connected to a central PC running Unity engine (2020.3.30f1) for the MR scene and data management. Participants stood approximately 1.5 m from the Azure Kinect to allow for a full-body capture.

#### 2.3.2. Data Processing and Statistical Analysis

For the SVV test, the angles of rotation of the whiteboard saved from Unity were evaluated to determine the angle of deviation from the vertical. SVV results were analyzed to determine angular deviations from the vertical, with deviations greater than ±2 degrees flagged as potential abnormalities. Normality of angular parameter distributions was verified using the Shapiro–Wilk test. Paired *t*-tests were selected to compare postural adjustments when reaching symmetrical targets (e.g., left vs. right), which reflect lateralized postural control strategies.

For the IDBA test, data collected from HL2 and Azure Kinect through Unity were elaborated to calculate the parameters: sway area of the head trajectory, velocity on the horizontal plane of the head, and the angles for knees and trunk. The trajectories of the head collected through HL2 were evaluated on the horizontal plane to visualize the sway area in the total time of the test. Trajectories were segmented into eight trajectories per target, between the instant in which the subject reaches the central ghost or the central ghost disappears and the instant in which the target is reached or disappears. This choice was due to the lack of clinical interest of the phase in which the subject returns to the central position. The time instants of the ghost disappearing or being reached were saved from Unity. The velocity on the plane, the angles for each target, and mean and standard deviation were calculated after the trajectories were cut into eight trajectories. Furthermore, statistical analysis was performed on IDBA results to identify the most significant parameters among the biomechanical angles identified as fundamental by medical experts, while using the proposed framework.

In particular, the analysis aimed at verifying if the trajectory followed by the patient’s CoP while performing Pac-Man exercises stimulated direction-specific postural strategies and balance-maintenance mechanisms, thereby challenging their limits of stability. This analysis was executed by comparing angular behaviors regarding diametrically opposite targets such as left vs. right or anterior vs. posterior. More specifically, a *t*-test was performed to compare the mean biomechanical angles obtained for targets positioned along the radial layout in diametrically opposite positions. Eventually, correlation between IDBA velocity and angular parameters was computed.

## 3. Results

### 3.1. SVV

As previously mentioned, the SVV test requires the acquisition of the angular inclinations of the white bar. At the end of the exercise, the inclination of the bar is supposed to be vertical. From the literature, any angular deviation from the vertical greater than ±2 degrees is considered a sign of pathology. As shown in Table 2, ten participants aligned the bar exactly at 0°, while five showed a 1° deviation. All deviations were within the normal clinical threshold (±2°), confirming task feasibility and sensitivity of the MR setup.

### 3.2. IDBA

**Figure 4:**
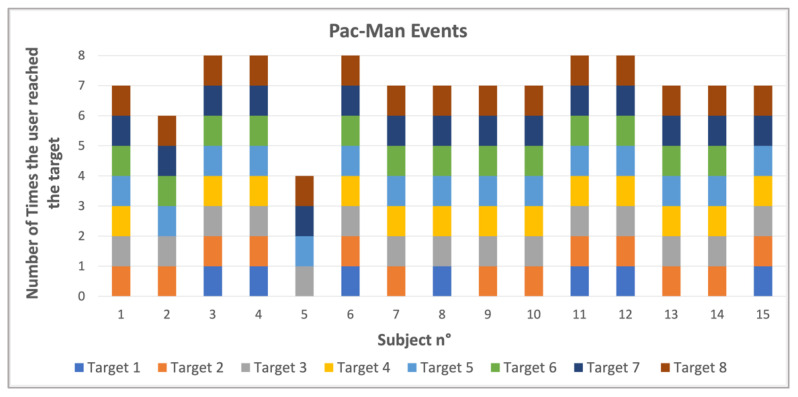
Number of times each target was reached and how many targets each subject reached.

**Figure 5:**
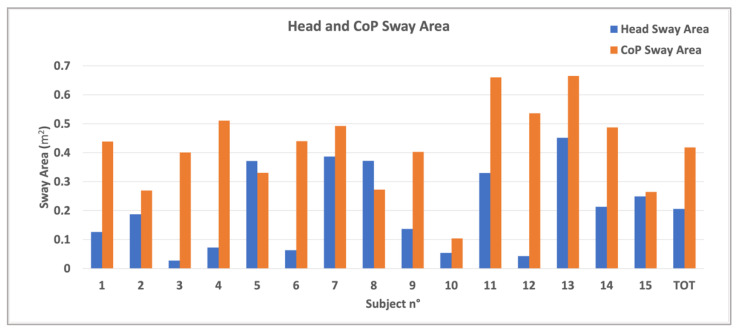
Sway area of the fifteen participants for both head (blue bar) and CoP (orange bar) and the mean values for all in the healthy group.

**Figure 6:**
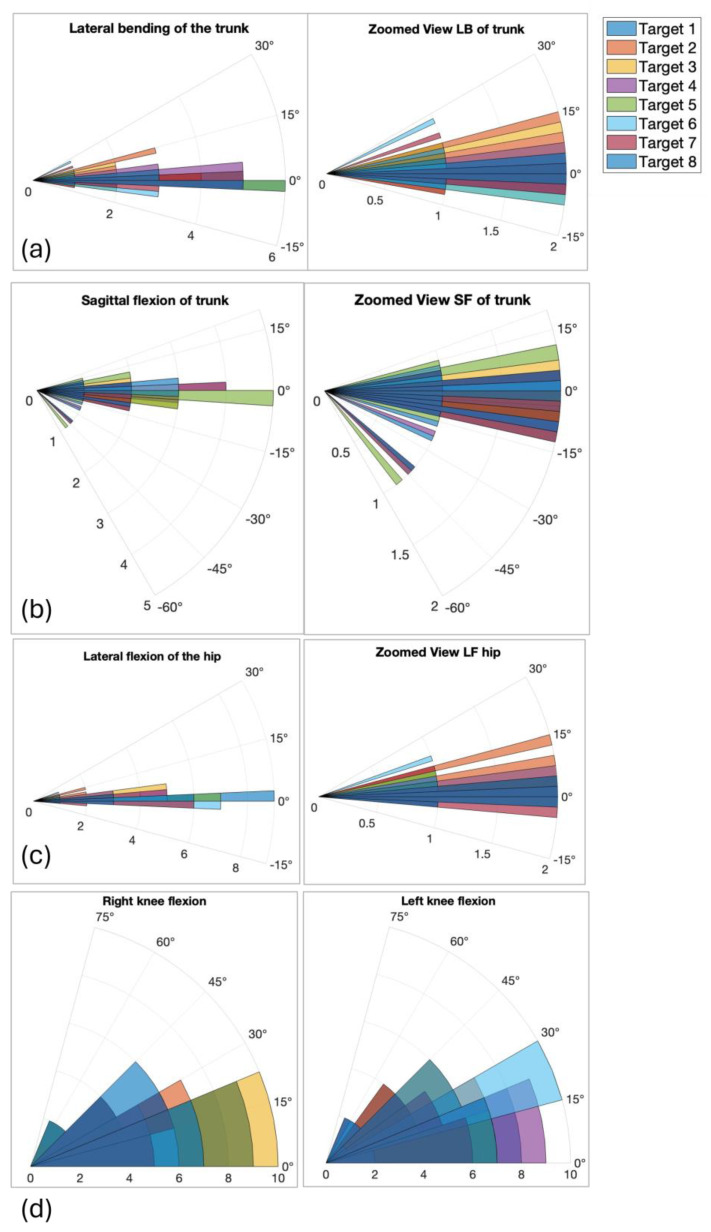
Polar plots showing mean angular deviations (deg) for trunk and hip oscillations across eight directional targets. Center represents 0 degrees deviation; radius reflects magnitude of flexion: (**a**) lateral blending of the trunk with the zoom view of the center of the graph (delta 5 degrees); (**b**) sagittal bending of the trunk with the zoom view of the center of the graph (delta 5 degrees); (**c**) lateral flexion of the hip with the zoom view of the center of the graph (delta 5 degrees); (**d**) right and left knee flexion (delta 15 degrees).

**Figure 7:**
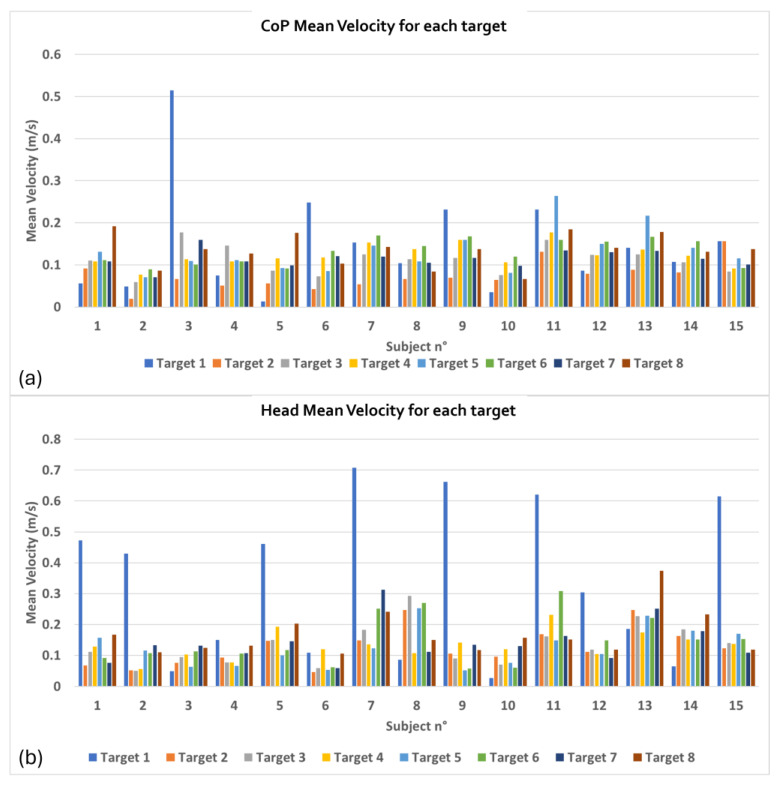
Average velocity required to the control group to reach targets: (**a**) CoP Mean Velocity for each target; (**b**) Head Mean Velocity for each target.

Figure 4, Figure 5, Figure 6 and Figure 7 show the output values of the parameters reported in Table 1 for all the members of the control group and for all eight targets. Figure 4 shows a comparative overview of how frequently each subject reached the different targets during the events. The 15 participants, labeled from 1 to 15, are represented on the *x*-axis. The *y*-axis represents the total number of targets the user reached and which target was reached.

Figure 5 shows the sway area of the fifteen participants for both head and CoP. The sway area is calculated based on the total duration of the acquisition of the test. In the graph, the mean values for the entire healthy group are also reported. Revised calculations corrected anomalous velocity values. The mean CoP velocity ranged from 0.10 to 0.45 m/s, while head velocity ranged from 0.05 to 0.20 m/s, consistent with expected movement in postural exercises. CoP sway area was significantly larger than head sway area (0.38 ± 0.15 cm^2^ vs. 0.15 ± 0.14 cm^2^, *p* < 0.001).

Figure 6 reports, in polar graphs, the mean values of angles measured from the Azure Kinect skeleton reconstruction, representative of the global posture each subject assumed during the test to reach the different targets. Although polar plots are not standard in the clinical balance literature, they were selected here to intuitively illustrate the directional dependence of postural responses. Each angle is plotted radially according to the corresponding target direction, allowing immediate visual comparison between movements toward different spatial positions. Figure 6a–c show the mean angular value of flexion angles of the trunk and hip on the sagittal and frontal plane, while Figure 6d shows the mean angles of knees flexion. Table 3 summarizes the mean and standard deviation of angular values among participants for each target.

**Figure 8:**
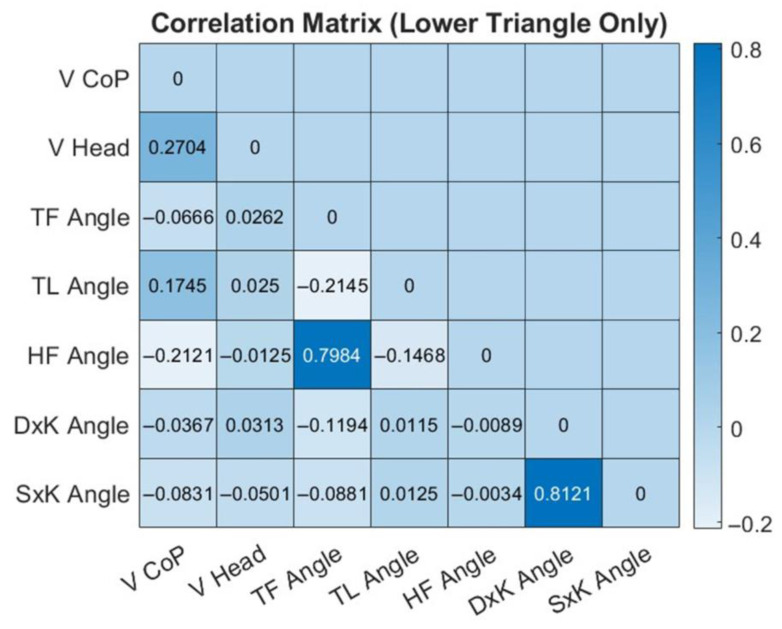
Heatmap of overall correlation indexes among angular and velocity parameters during the IDBA test. The color bar describes the strength and direction of correlation. Strong correlations include a high positive correlation between the TF Angle (trunk frontal angle) and the HF Angle (hip frontal angle) as well as the angular parameters of the right knee (DxK angle) and the left knee (SxK angle).

Postural angles showed directional dependence: significant differences (*p* = 0.01) in trunk and hip frontal oscillations were found between target 3 (right) and target 7 (left), indicating asymmetry in strategy. The polar plots include legends and axis labels, and Figure 8’s heatmap is simplified to show only significant correlations (e.g., trunk–hip, knee–knee).

Correlations were assessed among multiple joint parameters including pairs such as trunk–hip and knee–knee angular relationships. Table 3 shows the *p*-values derived from the *t*-tests performed on the angular parameters of the IDBA test. As previously mentioned, a statistical test was conducted on mean angular values obtained during attempts to reach opposite targets through CoP movements. Targets 3 and 7 are presented as a representative example of opposite lateral directions (right and left) where a statistically significant difference was observed. Other comparisons, such as between targets placed frontally and posteriorly (targets 1 and 5), did not show significant differences in angular parameters. This indicates that while the protocol is able to detect direction-specific variations in postural control, it is particularly sensitive to lateral movements involving distinct balance strategies.

The significant difference (*p*-value < 0.05) between trunk and hip frontal oscillations for targets 3 and 7 suggests that different motor control mechanisms are engaged depending on the direction of CoP displacement. This supports the protocol’s sensitivity in capturing meaningful changes in balance maintenance strategies when tasks involve lateral shifts, thereby validating its effectiveness for detecting direction-dependent postural adaptations.

Figure 7 shows the CoP (Figure 7a) and head (Figure 7b) average velocity on the horizontal plane measured to reach the targets. Bar graphs represent the average velocity values performed by each subject of the healthy group to reach each of the eight targets that appear during the exercise.

Figure 8 shows the correlation heatmap computed from the correlation indexes among the test’s angular and velocity parameters. Correlations were calculated both for each individual target and across the total dataset (15 participants’ attempts to reach 8 targets). Since the correlation tendencies were consistent along all targets, the reported heatmap is about the overall correlation.

## 4. Discussion

### 4.1. SVV

The results of the SVV test conducted with 15 healthy participants showed that most participants (10 out of 15) achieved an angular deviation of 0 degrees from the vertical, while the remaining 5 participants exhibited a 1-degree deviation. These findings are consistent with what is expected from a healthy control group, where deviations from the vertical are minimal. In the literature, any deviation greater than ±2 degrees is considered a potential indicator of pathology, suggesting that all participants in this test fall well within the normal range [10,26]. The results reinforce that the SVV test with MR can effectively assess verticality perception in healthy individuals, and the tool appears sensitive enough to detect even minor deviations, with no participants showing signs of pathology. Furthermore, since HL2 is equipped with IMU sensors, it allows for recording head tracking data, which is crucial to assess deviations from the vertical. Such data could broaden the research about the individual’s perception of vertical. The consistency of results with minimal deviation (median 0 degrees) further validates the accuracy and reliability of the system for clinical assessments of visual verticality. Even if other XR frameworks for SVV have already been proposed in the literature, such as the VR ones described in [27,28], the use of HL2 creates an MR scenario. MR does not isolate the user from the surrounding environment, as VR does, making the experience look more realistic. The lack of isolation also prevents motion sickness. Furthermore, if compared with possible AR prototypes, the user is given the possibility to physically interact with holographic buttons to move the SVV bar. Moreover, the test can be also built and uploaded on the device to make it independent from the computer. In this way, this technology provides the clinician with a user-friendly, small, and non-invasive device that allows the patient to perform the test autonomously and comfortably.

### 4.2. IDBA

The LOS test results provide several key parameters to evaluate the kinematics and postures of the participants using Azure Kinect, HL2, and WBB systems. While the combined framework may seem daunting, one may measure numerous parameters in real time. A set of variables such as this enables clinicians to measure and distinguish balance more precisely. The comparative value provided by the Pac-Man Events graph (Figure 4) illustrates the percentage of instances in which each subject hit a range of targets.

The findings suggest subject variability in the greater number of targets detected by some participants but fewer targets detected by others. This variability highlights the potential for using such dynamic tasks to assess and train balance and coordination. The sway area results (Figure 5) reveal that the CoP sway area is generally larger than the head sway area, meaning that the lower body tends to generate more sway during balance tasks. In fact, the scope of the exercise is to bring the CoP farther to the limit of the BoS. However, participants do not lean from the vertical in the erect position. Instead, they instinctively rotate and bring the hip around while they take the head near the center to balance the global body. This first result aligns with [29], in which the authors evaluate CoP and head sway in reaching different targets. They demonstrate that swaying is relevant to increase task performance and facilitate cognitive processing and that a correlation is possible between the head and CoP in a dynamic postural control. The blue and orange bar graphs demonstrate that sway remains within a controlled range for both head and hip in the control group, reinforcing the stability of healthy individuals during tasks. The differences in sway area between participants suggest variability in postural response, which could be useful in distinguishing between normal and pathological movement patterns in future research. In fact, as mentioned in [30], head motions can influence and make comparable effects on postural stability also in healthy participants.

The angles shown in Figure 6 support these interpretations. The angular measurements of trunk lateral and sagittal flexion indicate that participants exhibited varied movement patterns when attempting to reach different targets. While some targets required greater trunk movement, others performed more precise, controlled shifts, suggesting that target direction and distance influence body mechanics. To reach targets 1 and 5, which are on the vertical axis, some of the participants bend forward, while for targets 3 and 7, which are on the horizontal axis, some participants bend in the opposite direction of the target. Probably these situations are possible because these participants preferred moving the hip toward the target instead of moving the head and bending. For instance, lateral asymmetries in frontal oscillations may suggest dominant-side stabilization strategies or natural asymmetries of the participant. Looking at the angular flexions of both knees, participants mainly kept their legs extended (< 15 degrees), but, for targets on the vertical axis, they preferred to flex their legs and lean with the trunk while moving the hip. Knee flexion patterns were also indicative of how participants adjusted their lower body to maintain balance. This consideration was also made in [31], where the authors evaluated the stability of a group of healthy young women with different degrees of knee flexion. Meanwhile, [32] demonstrates that knee flexion, while lowering the hip in the vertical direction, limits the sway of the CoP on the BoS.

Finally, the head and CoP velocity data (Figure 7) highlight the varying degrees of effort required to reach each target. In general, the control group demonstrated consistency in the velocity values, which is indicative of efficient movement strategies in a healthy population. The velocity variation between targets shows that certain directions may be more challenging in balance maintenance, which can be further explored in clinical settings.

With its concurrent examination of CoP displacement and trunk and hip kinematics, the IDBA can identify subtle direction- and body-segment-dependent differences in balance maintenance strategies. For example, the sizable differences observed in trunk and hip oscillations in the frontal plane during lateral reaching suggest that different neuromuscular control mechanisms are at play, with possible clinical implications for the design of individualized rehabilitation interventions. This approach takes a step beyond demonstrating the potential for feasibility of CoP measurement with a low-cost device by illustrating the added clinical value resulting from the synthesis of complementary biomechanical parameters. Therefore, the novelty of the IDBA protocol is its ability to record and interpret dynamically the relationship between center of pressure behavior and segmental postural adjustments, creating a more informative and sensitive balance assessment tool.

Since this study aims to propose a new framework, the IDBA and SVV test currently have not been validated through comparison with gold-standard systems such as force platforms or optoelectronic systems. In a future stage, the accuracy of the system could be assessed through a comparison with existing systems such as Vision Lab Software 3.0 with a systematic error analysis. Further improvements will also include a test–retest study design as well as an inter-trial variance analysis as well as correction for multiple comparisons in *t*-tests across IDBA’s targets. Furthermore, the measurement of the subject’s individual maximum displacement before balance loss was not assessed in the feasibility stage. Thus, it does not adapt to each subject’s individual maximum displacement before balance loss. This choice is due to the creation of a uniform game condition for all participants, but future studies could integrate this feature for a user-specific balance assessment. Furthermore, a future comparison with a group of patients with balance disorders could be carried out to assess parameters and range values useful to discerning pathological from healthy participants. Eventually, since the framework has been tested only on young, healthy adults, its generalizability will be improved by study on clinical cohorts and older populations.

## 5. Conclusions

The current study stands as a proof of concept of the feasibility of the MR tool, especially via the integration of Azure Kinect and WBB, in comprehensively assessing balance and postural stability in healthy individuals through innovative tests. The SVV test results, with all participants showing deviations within the normal range (less than ±2 degrees), confirms the tool’s potential to identify deviations from vertical perception. During the IDBA test, the recorded measurements of trunk and knee angles and head and CoP velocities provided important insights into body mechanics and balance strategies during the target-reaching task. The relatively small range of head and CoP sway in all participants suggests that healthy individuals possess an extremely high degree of postural stability during exercise. However, these findings could be enhanced by taking a larger number of participants and by more research into the variability of some parameters. This XR system is utilized in a more experiential and interactive way, which can possibly lead to increased patient compliance and motivation toward rehabilitation interventions. The results of this study indicate that this XR-based system could be a promising tool for the clinical assessment of balance and motor control.

As stated in [1], it is important to enhance the conventional balance assessment methods with more precise, individualized approaches through the incorporation of sensor-based measurements. This approach thus aligns with the need for more personalized training feedback. By taking future research to populations with well-established comorbidities, especially to clinical population with balance disorders, this platform can potentially provide novel tools and means for the identification of related disorders and evaluation of improvements. Additionally, future verification in clinical settings could even extend its use to real-world clinical settings.

## Figures and Tables

**Table 1:** Parameters measured in IDBA test.

Parameter Name	Measurement	Device	Formula
Movement velocity in plane for CoP and Head (m/s)	Distance from the center covered by the CoP in the attempt toward the single target	WBB; HL2	$V=\left(\sum \sqrt{{\left(x_{i+1}-x_{i}\right)}^{2}+{\left(y_{i+1}-y_{i}\right)}^{2}}/\left(t_{i+1}-t_{i}\right)\right)/N$
Sway area for CoP and Head ($m^{2})$	Area of the ellipse fitted over the CoP or Head trajectory so that it contains 90% of all the data points	WBB; HL2	$SA=\;\pi \sqrt{{\left(x_{o}-x_{a}\right)}^{2}+{\left(y_{o}-y_{a}\right)}^{2}}\times \sqrt{{\left(x_{o}-x_{b}\right)}^{2}+{\left(y_{o}-y_{b}\right)}^{2}}\;$ where a and b axis of the ellipse
Angles of knee flexion (°)	Means and standard deviations of flexion angles of each knee	Azure Kinect	$Knee\;flexion=\arctan(z_{ankle}-z_{knee}/x_{ankle}-x_{knee})-\;\arctan(z_{hip}-z_{knee}/x_{hip}-x_{knee})$
Angles of trunk flexion and oscillation (°)	Means and standard deviations of flexion and oscillation angles of the trunk	Azure Kinect	$Oscillation\;on\;Sagittal\;Plane=arctan\left((z_{neck}-z_{pelvis})/\left(x_{neck}-x_{pelvis}\right)\right)$ Oscillation on Frontal Plane = $arctan\left((y_{neck}-y_{pelvis})/\left(x_{neck}-x_{pelvis}\right)\right)$

**Table 2:** Participants’ angular deviations resulting from SVV test.

Number of Participants	Angular Deviation (Degrees)
10	0
5	1

**Table 3:** *p*-values resulting from the *t*-test performed on mean angular values during the Pac-Man exercise. In particular, the statistical test was performed on angular values obtained during attempts to reach opposite targets through CoP movements. The table shows the results from statistical analysis conducted by comparing Target 3, placed on the subject’s right, with Target 7, on the participant’s left.

Angle	*p*-Value
Trunk frontal oscillation	0.0100
Trunk lateral oscillation	0.6117
Hip frontal oscillation	$8.474\times 10^{-4}$
Knee flexion Dx	0.9564
Knee flexion Sx	0.6656

## Data Availability

The data that support the findings of this study are available on request from the corresponding author. The data is not publicly available due to privacy restrictions.

## Supplementary Materials

The following supporting information can be downloaded at: https://www.mdpi.com/article/10.3390/bioengineering12080850/s1, Video S1: IDBA from user point of view; Video S2: IDBA from Unity engine; Video S3: SVV from user point of view.
